# Degradative actions of microbial xylanolytic activities on hemicelluloses from rhizome of *Arundo donax*

**DOI:** 10.1186/s13568-014-0055-6

**Published:** 2014-07-09

**Authors:** Licia Lama, Annabella Tramice, Ilaria Finore, Gianluca Anzelmo, Valeria Calandrelli, Eduardo Pagnotta, Giuseppina Tommonaro, Annarita Poli, Paola Di Donato, Barbara Nicolaus, Massimo Fagnano, Mauro Mori, Adriana Impagliazzo, Antonio Trincone

**Affiliations:** 1 Institute of Biomolecular Chemistry ICB-CNR, Consiglio Nazionale delle Ricerche, Via Campi Flegrei 34, Pozzuoli, 80072, Napoli, Italy; 2 Department of Environmental Sciences, University of Naples “Parthenope”, Centro Direzionale, Isola C4, Naples, 80143, Italy; 3 Department of Agriculture, Naples University Federico II, Via Università 100, Portici, 80055, Naples, Italy

**Keywords:** Biorefinery, Xylan, Thermophilic enzymes, Xylanases, Xylooligosaccharides, Arundo donax

## Abstract

Polysaccharidases from extremophiles are remarkable for specific action, resistance to different reaction conditions and other biotechnologically interesting features. In this article the action of crude extracts of thermophilic microorganisms (*Thermotoga neapolitana*, *Geobacillus thermantarcticus* and *Thermoanaerobacterium thermostercoris*) is studied using as substrate hemicellulose from one of the most interesting biomass crops, the giant reed (*Arundo donax* L.). This biomass can be cultivated without competition and a huge amount of rhizomes remains in the soil at the end of cropping cycle (10–15 years) representing a further source of useful molecules. Optimization of the procedure for preparation of the hemicellulose fraction from rhizomes of *Arundo donax*, is studied. Polysaccharidases from crude extracts of thermophilic microorganisms revealed to be suitable for total degradative action and/or production of small useful oligosaccharides from hemicelluloses from *A. donax*. Xylobiose and interesting tetra- and pentasaccharide are obtained by enzymatic action in different conditions. Convenient amount of raw material was processed per mg of crude enzymes. Raw hemicelluloses and pretreated material show antioxidant activity unlike isolated tetra- and pentasaccharide. The body of results suggest that rhizomes represent a useful raw material for the production of valuable industrial products, thus allowing to increase the economic efficiency of *A. donax* cultivation.

## Introduction

Among interesting thermostable enzymes isolated from microorganisms, polysaccharidases are remarkable for specific action, resistance to different reaction conditions and other biotechnologically interesting features. Microorganisms of the genus *Thermoanaerobacterium* and others of thermophilic or hyperthermophilic origin, are relevant for their abilities to bioconvert carbohydrates. In the framework of a research project focused on bioprospecting for thermophilic microorganisms, a new species, *T. thermostercus* (designated as Buff), was recently isolated from buffalo-dung and classified as a new member of *Thermoanaerobacterium*. The optimum growth temperature, pH and NaCl concentration were, respectively, 60°C, 6.5 and 0.5% (w/v). As carbon sources this strain utilized glucose, mannose, xylose, maltose, cellobiose, sucrose and xylan (Romano et al. [2010]). *Geobacillus thermantarcticus*, a thermophilic bacterium isolated from Antarctic geothermal soil near the crater of Mount Melbourne, produced extracellular xylanase (1,4-β-D-xylan xylanohydrolase; E.C. 3.2.1.8) and β-xylosidase (1,4-β-D-xylan xylohydrolase; E.C. 3.2.1.37); the studied action of these two enzymes on commercial xylan gave only xylose (Lama et al. [2004]). Glycosyl hydrolases in *Thermotoga neapolitana* resulted interesting in the enzymatic process for the cleavage of xylan to smaller oligosaccharides and also in their coupling to aglycones by transglycosylating activity achieved under certain reaction conditions (Tramice et al. [2007]).

In biomass refinery, lignocellulosic material is fractionated to obtain the main components (cellulose, hemicelluloses, and lignin). Xylan is the major hemicellulosic component. Polysaccharidic chain is composed by a backbone of β-1,4-xylose units in pyranose form, decorated on different position with 4-O-methyl-D-glucuronic acid and L-arabinofuranose units. This skeleton can also be extensively esterified with acetic or ferulic acids; the latter in some species, provides a chemical link between hemicellulose and lignin. D-xylose and L-arabinose as end products are derived by biorefinery enzymatic processes from xylan of lignocellulosic biomasses. However, up until recently biomass-to-fuel investigation has neglected heteroxylan from lignocellulosic. This has been attributed to a number of reasons pointing out that (i) a sub-optimal settings for material extraction or (ii) the slow development of enzymatic processes and (iii) the scarce exploitation of derived products, characterize the field of pentose-specific conversion/utilization technologies. Specific xylanase actions on xylan could lead to important oligosaccharides (Dumon et al. [2012]). Interesting bioactivities have been reported for both poly- and oligosaccharidic fragments. Immunostimulating effects and anti-inflammatory and antimicrobial activities have been reported for arabino(glucurono)xylans isolated from different sources (Christakopoulosa et al. [2003]). A nutraceutical perspective has been also considered for these compounds (Saeed et al. [2011]). A gastroprotective arabinoxylan from sugarcane bagasse was isolated (Mellinger-Silva et al. [2011]). A structure-activity relationship study on the role of 4-O-methyl glucuronic acid (MeGlcUA) in regulating aggregation of β-polyxylosides of (9H-fluoren-9-yl)-methanol obtained via the transglycosylating action of *Thermotoga neapolitana* β-xylanase, was also studied. An anti-proliferative test of these compounds on human epithelial EFO 27 ovarian cancer cells indicated that the presence of MeGlcUA modulates biological activity, while its absence induced molecular aggregation (Tramice et al. [2009]).

With the increasing demand of ligno-cellulosic materials in processes such as co-generation, production of biofuel and compost from municipal solid wastes, etc., a competition for land used for food production is observed with potential risks for food security in wide areas of the world (van der Horst and Vermeylen [2011]). Among the different biomass crops for ligno-cellulosic materials, one of the most interesting is giant reed (*Arundo donax* L.), because it is tolerant to a wide range of environmental stresses so that it can be cultivated on marginal, degraded or contaminated lands thus reducing competition with food crops which generally require a better quality arable land (Di Nasso et al. [2013]; Fiorentino et al. [2010]; Diodato et al. [2009]). *Arundo donax* is suggested in projects (i.e. Ecoremed 2011. LIFE11/ENV/IT/275), (Fagnano [2014]) of phytoremediation of polluted soils (Bonanno et al. [2013]). The most studied part of *Arundo donax* is represented by shoots (reeds) of interest in bioethanol, biodiesel (Pirozzi et al. [2010]) or biopolymer production (Galletti et al. [2013]). However at the end of cropping cycle of a giant reed stand (10–15 years), a huge amount of rhizomes remains in the soils and they have to be eliminated to afford further agricultural utilizations. These rhizomes could represent a further source of useful molecules for bioenergy or biochemicals (Di Nasso et al. [2013]).

In the present article an overview of degradative actions of different enzymatic extract on hemicelluloses from rhizomes of *Arundo donax* is presented, comparing the processes acted by mesophilic and thermophilic biocatalysts. Convenient set up of a degradative processes with/without production of small useful oligosaccharides are reported.

## Methods

### General

Total carbohydrate content was determined by Bernfeld ([1955]) method using a xylose-based calibration curve. Xylose was determined by D-xylose assay kit (Megazyme), specifically designed to measure this sugar in samples containing up to 5 mg of glucose in the final assay reaction without any interference. Glucose was assayed enzymatically by GOPOD-FORMAT, Megazyme. Protein concentration was determined by the method of Bradford ([1976]) using bovine serum albumin as the standard. *Thermomyces lanuginosus* xylanase, (X2753 2500 U/g) was obtained from Sigma. *Thermotoga maritima* endo-1,4-β-xylanase, 22 U/mg of protein (wheat arabinoxylan at pH 5.0 and 40°C) was obtained from Megazyme.

^1^H NMR spectra were recorded at 600.13 MHz on a Bruker DRX-600 spectrometer, equipped with a TCI Cryo Probe TM, fitted with a gradient along the Z-axis. Spectra in D_2_O were referenced to internal sodium 3-(trimethyl-silyl)-(2,2,3,3-2H4) propionate (Aldrich, Milwaukee, WI). ^13^C NMR, JMOD-^1^H and COSY, TOCSY, HSQC, HSQC-EDITED, HMBC (^3^ J: 7 and 10 Hz), NOESY (mixing time at 100, 200, 300 msec) experiments were used for structural determination.

Mass spectra were acquired on a microQ-Tof mass spectrometer coupled with an Alliance HPLC (Waters, Milford, MA) equipped with an ESI source.

### Microorganism and culture conditions

*Geobacillus thermantarcticus* (DSM 9572) isolated from Antarctic geothermal soil was grown in a flask (2 L) at 60°C on a medium containing xylan at pH 6.0 as previously reported (Nicolaus et al. [1996]). *Thermoanaerobacterium thermostercoris* strain BUFF was grown at 60°C in a 40 L fermentor (B. Brown Biotech International Micro DCU400) as previously reported (Romano et al. [2010]). *Thermotoga neapolitana* DSM 4359 obtained from DSMZ (Deutsche Sammlung von Mikroorganismen und Zellkulturen) was grown at 80°C in a 40-L fermentor (B. Brown Biotech International Micro DCU400) as previously reported (Tramice et al. [2007]).

Microbial growth was monitored turbidimetrically at 540 nm and cells were harvested in late exponential growth phase and collected by continuous centrifugation using an Alpha-Laval centrifuge and then pelleted by centrifugation at 10,000 rpm for 30 min and frozen.

### Preparation of crude enzymatic extracts

*Thermoanaeorbacterium thermostercoris* (CFE-TT protein 11.4 mg/ml), and *Thermotoga neapolitana* cell free extracts (CFE-TN protein 34.4 mg/ml) were prepared in acetate buffer 50 mM pH 5.5 disrupting the cells by usual techniques (freeze-thawing, lysozyme 100 μg/ml). *Geobacillus thermantarcticus* extracellular suspension (ES-GTM1, protein 0.37 mg/ml) was prepared by adding ammonium sulfate to the cell-free broth (1 L) to 80% saturation. The precipitate was recovered by centrifugation (10,000 g, 1 h, 4°C), dissolved in 50 mM sodium acetate buffer (pH 5.6) and dialyzed overnight against the same buffer.

### Biomass

*Arundo donax* was grown under Mediterranean climatic conditions: during the growth period (April-November 2012) rainfall was 355 mm and Reference Evapotranspiration was 923 mm and thus water deficit was very severe (568 mm). Rhizomes of *A. donax* were harvested at the experimental farm of the University “Federico II” Naples and were obtained from a 4 year experimental plantation located in the Sele river plain of Campania Region (Southern Italy, 40°61’N, 14°92’E, 30 m a.s.l.). Rhizome samples were harvested in a sample area of 2 m^2^, and then chipped, dried at 60°C until constant weight and grinded at 4 mm. Successively, the material was grinded in order to obtain a homogenous fine powder (particle size of about 1 mm) by means of a kitchen mixer (Waring).

### Preparation and optimization of hemicellulosic substrates from rhizome of *Arundo donax*

The hemicellulose fraction was solubilized in alkali medium by continuous magnetic stirring at room temperature; different 2% w/v solutions of rhizome of *Arundo donax* in potassium hydroxide (KOH, 0.5 N, 1 N and 2 N) were used at different time intervals, 24 h, 48 h, 72 h and 96 h. Experiments for the optimization of extraction procedure were conducted in triplicate; briefly, the grinded dry material was suspended in KOH solutions; afterwards the suspension was sieved (0.5 mm screen) and centrifuged at 10,000 rpm for 40 min at 4°C. The supernatant was treated with same volume of cold ethanol 96% (v/v) added drop wise under stirring. The alcoholic suspension was stored at −20°C overnight and then centrifuged at 10,000 rpm for 1 h at 4°C. The pellet was solubilized in hot distilled water, cooled at room temperature, the pH was adjusted to 7.0 with HCl and dialyzed against distilled water using Spectrapore dialysis tubes (12,000-14,000 MW cut-off), and finally freeze-dried. The lyophilized material (RK72h, RK48h, RK24h) was weighed using a Mettler Toledo analytical balance and the yield was expressed in weight (%, w/w) with respect to the initial dry biomass.

### Enzymatic reactions

Hydrolysis reactions were conducted using various biocatalysts and hemicellulosic material prepared from *A. donax* (RK72h) as follows: *Thermomyces lanuginosus* xylanase, 22 units were dissolved in 3 ml phosphate buffer 50 mM, pH 7 containing 30 mg substrate. Reaction was performed at 34°C under stirring for 26 h and stopped by enzyme denaturation at 100°C (2 min). *Thermotoga maritima* endo-1,4-β-xylanase, 57 units were dissolved in 1 ml in acetate buffer 50 mM, pH 5.6 containing 10 mg of substrate. Reaction was performed under stirring at 70°C, 24 h and stopped by cooling. Cell free extract (CFE-TT) from *Thermoanaeorbacterium thermostercoris* (proteins: 11.4 mg/ml), was added to the needed amount of substrate (4 mg/ml of reaction) in acetate buffer 50 mM, pH 5.6. Reaction was performed at 50°C for 24 h under stirring. Cell free extract (CFE-TN) from *Thermotoga neapolitana* (proteins: 34.4 mg/ml), was added to the needed amount of substrate (4 mg/ml of reaction) in acetate buffer 50 mM, pH 5.6. Reaction was performed at 90°C for 24 h under stirring. Extracellular suspension of *Geobacillus thermantarcticus* 8 ml, ES-GTM1b, (proteins: 0.37 mg/ml) was reacted with 40 mg of substrate solubilized in 2 ml phosphate buffer 50 mM, pH 6, at 70°C for 24 h. In a different condition, ES-GTM1a, 1.5 ml were reacted with 20 mg of substrate solubilized in 4 ml phosphate buffer 50 mM, pH 6, or at 70°C for 24 h.

Transglycosylation reactions were performed using various biocatalysts as follows: 20 mg of *A. donax* (RK72h) in 1 ml of appropriate buffer/acetonitrile (87:13 v/v) containing 19.6 mg of 9-fluorene methanol; the reactions started by adding the different biocatalysts (57 U *Thermotoga maritima* endo-1,4-β-xylanase, at 70°C, 24 h; 500 μL *Thermotoga neapolitana* cell free extract (CFE-TN) at 70°C, 24 h; 500 μL cell free extract (CFE-TT) from *Thermoanaeorbacterium thermostercoris* at 50°C, 24 h; 4 ml of extracellular suspension *Geobacillus thermantarcticus* (ES-GTM1) at 70°C, 24 h) under stirring.

### Monitoring of reactions, purification and analysis of products

Hydrolyses reactions were monitored by TLC in solvent A: n-BuOH/AcOH/H_2_O 6:2:2 v/v, or B: EtOAc/AcOH/2-propanol/HCOOH/H_2_O, 25:10:5:1:15 by vol, whereas transglycosylation reactions were monitored in solvent C: EtOAc/MeOH/H_2_O 70:20:10 by vol. Chemical determination of reducing sugars and enzymatic determinations of glucose and xylose were conducted in triplicate. Hydrolysis reaction mixture was concentrated under vacuum and loaded onto a Biogel P-100 column (1 × 35 cm) equilibrated with water and eluted at 0.1 ml/min with water. A second Biogel P-2 column (1 × 47 cm) was performed eluting at 0.3 ml/min with water. The fractions containing the carbohydrates were pooled and freeze-dried. Purified material was subjected to NMR spectroscopy. The reactions of transglycosylation were stopped by cooling and immediately subjected to reverse-phase column chromatography (Merck Lobar RP-18) eluting with water, and then with methanol thus efficiently separating total chromophoric xylosylated fraction and unreacted acceptor in methanol fraction from free saccharides in water. The methanolic fractions were collected and analysed by ESI-MS.

Monosaccharides composition of *Arundo donax* polysaccharides was determined by GC-MS of acetylated methyl glycosides (Silipo et al. [2012]). Methanolysis was performed in 1.25 M HCl in methanol. In brief, sample (1.5 mg) was solubilized in anhydrous 1.25 M HCl/MeOH (1 ml) and then heated at 80°C overnight. Subsequently, air-dried samples were acetylated with acetic anhydride (50 μl) and pyridine (50 μl) at 100°C for 30 min. Then, samples were dried and washed with methanol in order to evaporate pyridine and dissolved in acetone (400 μl) and 1 μl was used for GC-MS analysis. All GC-MS runs were conducted on a Hewlett-Packard Series II Plus gas chromatograph (column: 30 m × 0.25 mm HP-5MS Hewlett-Packard Co.) coupled with a Hewlett-Packard 5989B mass spectrometer. Electron voltage was set at 70 eV and He was used as gas carrier at a costant flow rate of 1 ml/min. The temperature program used was 150°C for 3 min; from 150 to 280°C at 10°C/min; 280°C for 20 min.

### Activity assays

Antimicrobial activity was carried out using liquid culture of *E. coli* (DSM 498), *B. subtilis* subsp. *spizizenii* (DSM 347), and *M. luteus* (DSM 348). The MIC was determined by a serial dilution, in duplicate, starting from 100 μg/mL to 0.01 μg/mL. Bacterial and yeast growths were observed after 48 h of incubation. Cytotoxic activity was evaluated by the brine shrimp (*Artemia salina*) test in triplicate with appropriate amounts of samples dissolved in DMSO (1% final volume) to reach final concentrations of 100, 10 and 1 μg/ml, using 10 freshly hatched larvae suspended in 5 ml of artificial seawater (Meyer [1982]). Briefly, for each dose tested, surviving shrimps were left at RT and counted after 24 h, and the data statistically analyzed by the Finney program (Finney [1971]), which affords LD_50_ values with 95% confidence intervals.

Free-radical scavenging activity was evaluated on pre-treated grinded biomass in DMSO and on RK72h dissolved in H_2_O at a concentration of 20 mg/mL, and assayed for DPPH test (Blois [1958]). Different amounts (10, 50, 100 and 250 μL) of these solutions were added to 0.7 mL of DPPH in MeOH (6 mg/50 mL; 0.1 mM final concentration) and adjusted to 2 mL final volume with MeOH. The absorbance at 517 nm was determined after 30 min at room temperature and the percent of free radical inhibition was calculated. Fucoidan (Sigma) was used as standard. Free radical scavenging activity of samples was estimated as % inhibition of free radical DPPH. Antioxidant activity of both samples was also determined by DMPD method (Fogliano et al. [1999]). The reaction mixture contained 1 mM DMPD, 0.1 mM ferric chloride in acetate buffer 0.1 M (pH 5.25) in a total volume of 1 mL. The assay temperature was 25°C. The reaction was monitored at 505 nm until absorbance became stable at a value of 0.900 ± 0.100. Samples were dissolved in H_2_O at a concentration of 20 mg/mL. According to the method, the antioxidant activity of samples was carried out in triplicate on main solution (20 mg/mL) and on its diluted solutions 1:2, 1:5, 1:10. Then, 250 μL of solutions were added to the reaction mixture and the decrease in absorbance, which is proportional to the DMPD^+^ quenched, was determined after 20 min at room temperature. Fucoidan was used as standard. The antioxidant activity was reported as % inhibition of radical cation DMPD.

## Results

### Hemicellulosic substrates from rhizomes of *Arundo donax*: preparation and optimization of the procedure

Aboveground biomass yield of *Arundo donax* was 39.5 ± 8.1 t/ha and rhizome yield was 40.0 t/ha. With this root:shoot ratio of 1 the results of other researches carried out in Mediterranean conditions on mature giant reed stands (Di Nasso et al. [2013]), were confirmed.

**Figure 1:**
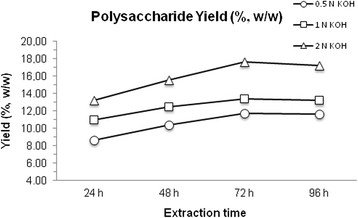
**Optimization of polysaccharide extraction from** ***A. donax*** **rhizomes.** Experiments were performed in triplicate with lyophilized material used in appropriate amounts; data are means of the values obtained that did not differ by more than 2%. Yield was expressed in weight (%, w/w) with respect to the initial dry biomass. See Methods for details.

Methods for the isolation of hemicelluloses from biomass include extraction in alkaline, alkaline peroxide and liquid hot water medium or steam explosion-based extraction, and other procedures based on slight modifications (Peng et al. [2012]; Verbruggen et al. [1995]). Under alkaline solubilization of hemicellulosic components, the native structure of xylan is extensively modified with saponification of ester-linkages and swelling of cellulose. Results of the optimization of the procedure for preparation of the hemicellulose fraction from rhizomes of *Arundo donax*, are reported in Figure 1.

The material extracted with 2 N KOH for 72 h at room temperature (RK72h) represented the 17.6% (w/w) of the dried biomass (Figure 1). In Table 1 is reported the monosaccharide composition of the extracted material*.* Arabinose to xylose ratio (Ara/Xyl) has been considered indicative of the degree of linearity or branching of hemicelluloses (Verbruggen et al. [1995]). The arabinose/xylose ratio of polysaccharide extracted from rhizomes of *A. donax* was 0.12, thus implying that the hemicelluloses obtained occurred in a partially de-branched form. ^13^C NMR spectrum (saturated solution, *d*-DMSO 40°C) of hemicellulosic substrate, RK72h, showed intense signals due to xylan main chain at 101.7, 75.3, 73.9, 72.5, 63.2 ppm, typical values of C1 (101.7) and C4 (75.3) linked β-xyloses. Small signals are present in accord to the monosaccharide composition (Table 1), in particular the percentage of arabinose paralleled in the NMR spectra to typical C1 (107.1) and others (87.3, 86.0, 80.2, 77.8, 61.8 ppm) signals of α-Ara*f* and C1 (103.5) signals of β-glucose.

**Table 1:** **Monosaccharide composition of** ***Arundo donax*** **polysaccharides extracted with KOH 2 N at 72 h**

**Monosaccharide**	***Arundo donax *%**
**Xyl**	57.13
**Glc**	34.77
**Ara**	7.03
**Man**	0
**Gal**	0
**Rha**	0
**GalA**	0
**GlcA**	1.07

*Abbreviations*: *Xyl* Xylose, *Glc* Glucose, *Gal* Galactose, *Ara* Arabinose, *Rha* Rhamnose, *Man* Mannose, *GlcA* Glucuronic acid, *GalA* Galacturonic acid.

### The action of crude homogenates on rhizomes of *Arundo donax*

The action of crude extracts obtained from *Thermotoga neapolitana* CFE-TN, *Geobacillus thermantarcticus* ES-GTM1 and *Thermoanaerobacterium thermostercoris* CFE-TT has been compared to the action of known commercial xylanases, *Thermomyces lanuginosus* and *Thermotoga maritima*, to study a convenient set up for the degradative action with production of small useful oligosaccharides from hemicellulose fraction of *Arundo donax*. Reaction products were used as candidates in bioactivity assays. Analysis of enzymatic action was conducted comparing (i) total conversion (total yield of reducing sugars and xylose and glucose percentages of reaction mixture, Table 2), (ii) TLC patterns of reaction products and (iii) NMR results.

**Table 2:** **Degradative actions of microbial and commercial xylanolytic activities on** ***A. donax*** **biomass and transglycosylation reactions**

	**Biocatalyst**	**S/B**	**RS%**	**Xyl%**	**Glc%**	**Useful results**
1	TL-xylanase	3.3	23.5	2.26	8.6	xylobiose 3.3 g/L
						pentasaccharide 1 (Figure 2), 2 g/L
2	TM-xylanase	2.6	38	0	0	-
3	CFE-TT	3.5	90	19.2	16.9	-
4	CFE-TT	1.2	100	28.6	6.8	-
5	CFE-TT	3.5	20	8.7	7.8	tetrasaccharide 2 (Figure 2) 2.64 g/L
6	CFE-TN	1.2	75	40.5	15.2	see Figure 3
7	ES-GTM1b	13.5	73.5	26.5	0	see Figure 3
8	ES-GTM1a	36.3	63.1	9.85	0	see Figure 3
			Comment			
9	TM-xylanase	7.7	Formation of mono and dixylosylated acceptor			
10	CFE-TN	1.2	Formation of mono and dixylosylated acceptor			
11	ES-GTM1	13.5	Formation of mono, di and trixylosylated acceptor			
12	CFE-TT	3.5	Formation of mono and dixylosylated acceptor			

Data are expressed in percentages using means of the values obtained by chemical and enzymatic determinations conducted in triplicate that did not differ by more than 5%. S/B = amount of biocatalyst, mg substrate/mg total protein; RS = Reducing sugars yield % (DNS); TL = *Thermomyces lanuginosus* xylanase; TM = *Thermotoga maritima* xylanase; CFE-TT *=* cell free extract of *Thermoanaeorbacterium thermostercoris*; CFE-TN = cell free extract of *T. neapolitana*; ES-GTM1 = extracellular suspension *Geobacillus thermantarcticus*.

**Figure 2:**
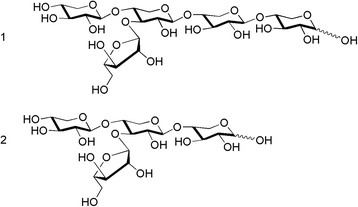
**The pentasaccharide β-D-Xyl** ***p*-(1–4)-[α-L-Ara** ***f*-(1–3)]-β-D-Xyl** ***p*-(1–4)-β-D-Xyl** ***p-*** **(1–4)-β-D-Xyl** ***p*** **(1) from hydrolysis mixture using** ***T. lanuginosus*** **enzyme and β-D-Xyl** ***p*-(1–4)-[α-L-Ara** ***f*-(1–3)]-β-D-Xyl** ***p*-(1–4)-D-Xyl** ***p*** **(2) the tetrasaccharide, from the hydrolysis reaction mixture using crude extract of** ***T. thermostercoris.***

The reaction of endo-β-(1–4)-xylanase from *Thermomyces lanuginosus* with *A. donax* biomass (Table 2, entry 1) was stopped at 26 h when 23.5% total reducing sugars were formed; values for free xylose and glucose indicated small presence of these two free monosaccharides. Indeed, after purification of reaction mixture, 3.3 g/L of xylobiose, identified by NMR spectroscopy, and 2 g/L of an interesting pentasaccharide (1, Figure 2), were obtained. The pentasaccharidic nature of this compound was quickly evidenced by the peak at (m/z: 701 [M + Na^+^] in the mass spectrum. ^1^H and ^13^C NMR chemical shift values are reported in Table 3, they secured that the compound was constituted by four units of β-xylose 1,4 linked (Xyl_1_ to Xyl_4_ starting from non-reducing end) and one unit of α-L-arabinose. In the ^1^H-NMR spectrum there were four anomeric signals of β-Xyl*p* at 4.474, 4.371, 4.330 and 4.406 ppm and one at 5.284 ppm indicating the presence of a *α-*L-Ara*f*. Positioning of *α-*L-Ara*f* was achieved by evaluating long-range correlations in HMBC experiments; in fact anomeric carbon of arabinose at 108.54 ppm correlated with proton signal at position 3 (3.65 ppm) of Xyl_2_ unit. Long-range correlations were also recorded between carbon signal of Xyl_2_ at position 4 and the anoneric proton of Xyl_1_ (1, Figure 2). In NOESY experiments, interglycosidic NOE contacts were recorded between Xyl_2_ anomeric proton (4.406 ppm) and the signal at 3.678 ppm of proton 4 of Xyl_3_ and between anomeric proton signal at 4.37 ppm of Xyl_3_ with the signal at 3.667 ppm, proton 4 of Xyl_4_. All remaining chemical shift values of the pentasaccharide 1, (Figure 2), were in agreement with spectroscopic data of similar structure reported in literature (Pastell et al. [2008]; Vliegenthart et al. [1992]).

**Table 3:** ^**1**^**H and**^**13**^**C chemical shifts of compounds in Figure**2

	**1**		**2**		**3**		**4**		**5**	
**1 Figure**2	^**1**^**H**	^**13**^**C**	^**1**^**H**	^**13**^**C**	^**1**^**H**	^**13**^**C**	^**1**^**H**	^**13**^**C**	^**1**^**H**	^**13**^**C**
α-L-Ara*f*	5.289	108.54	4.055	81.63	3.795	78.3	4.164	85.90	3.684	62.36
									3.617	
1: β-D-Xyl*p*	4.330	**102.49**	3.142	74.35	3.304	76.61	3.480	70.19	3.806	66.14
									3.180	
2: β-D-Xyl*p*	4.406	102.67-102.69	3.331	74.68-74.59	**3.650**	**77.40**	**3.710**	**74.68-74.59**	4.016	63.84-64.00
									3.950	
3: β-D-Xyl*p*	4.371	102.67-102.69	3.195	73.70-73.96	3.440	75.09-74.93	**3.678**	**77.40**	4.016	63.84-64.00
									3.290	
4: α,β-D-Xyl*p*	β: 4.474	97.53	3.142	74.35	3.441	75.09-74.93	**3.667**	**77.40**	3.950	63.84-64.00
	α: 5.070	93.02							3.280	
	**1**		**2**		**3**		**4**		**5**	
**2 Figure**2	^**1**^**H**	^**13**^**C**	^**1**^**H**	^**13**^C	^**1**^**H**	^**13**^**C**	^**1**^**H**	^**13**^**C**	^**1**^**H**	^**13**^**C**
α-L-Ara*f*	5.321	108.52	4.070	81.63	3.817	78.26	4.187	85.74	3.711	62.29
									3.635	
1: β-D-Xyl*p*	4.356	**102.40**	3.166	74.55	3.330	74.40	3.511	70.19	3.839	66.17-66.07
									3.190	
2: β-D-Xyl*p*	4.428	102.57	3.361	74.30	**3.658**	**77.39**	**3.697**	**76.78**	3.281	66.17-66.07
									3.975	
3: α,β-D-Xyl*p*	α: 5.115	92.97	3.175	74.25	3.460	75.00	**3.741**	**76.52**	4.049	63.95-61.93
	β: 4.496	97.58							3.315	

Indicative signals for interglycosidic linkage assignments are indicated in bold.

In a similar hydrolysis reaction (Table 2, entry 2) conducted using xylanase from *T. maritima* the production in high amount of xylobiose and small xylooligomers as main products from *Arundo* biomass, was confirmed. The absence of glucose was due to better purity of enzymatic activity (xylanase).

**Figure 3:**
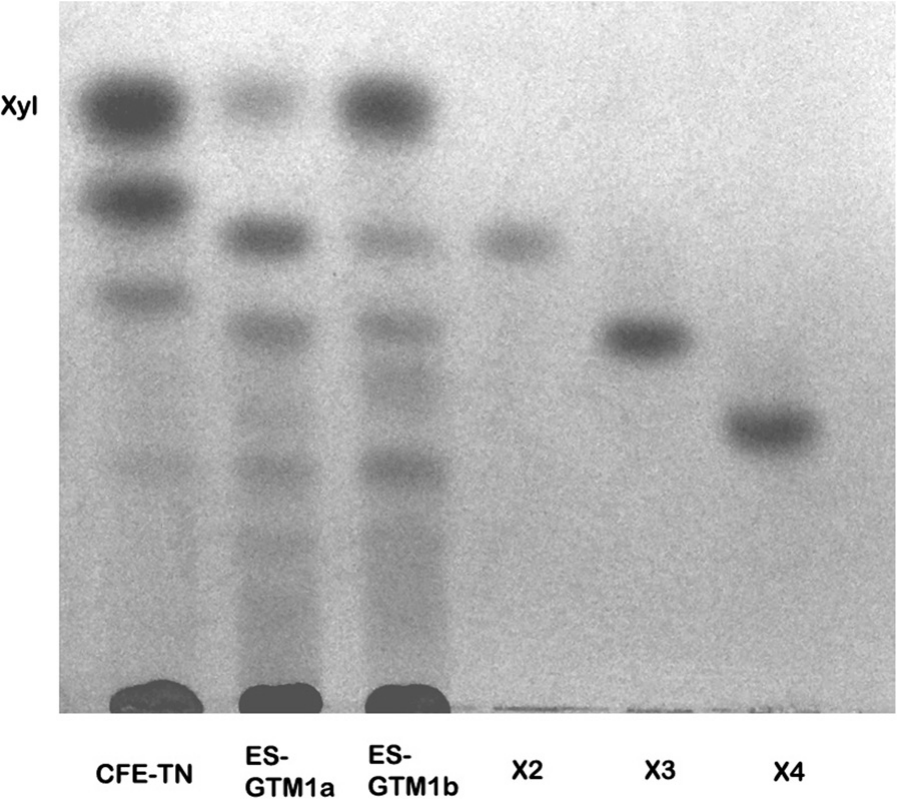
**TLC pattern of enzymatic reactions using** ***A. donax*** **hemicelluloses extract as substrate.** CFE-TN = cell-free extract *T. neapolitana* (Table 2, entry 6); ESGTM1a and ESGTM1b = extracellular suspension of *Geobacillus. thermantarcticus* (Table 2, entry 7 and 8); *X*2-X4 = β-1,4-xylooligomers.

The analysis of reactions (Table 2, entries 3–5) using cell-free extracts from *T. thermostercoris* and *T. neapolitana* (Table 2, entry 6) indicated high conversions of the polysaccaridic constituent in terms of reducing sugars (entries 3,4 and 6). The presence of β-xylosidases and xylanase activities in these enzymatic preparations is evidenced by the formation of high amount of free xylose. Low amounts of different xylose tetra- and pentaoligomers were found at the proper Rf in TLC in the course of reaction. In addition, as evidenced by glucose enzymatic determination, substantial amounts of glucose are formed proving the presence of interesting β-glucosidases (cellulase) activities (entries 3–6, Table 2 and Figure 3).

The reaction using *T. thermostercoris* was conducted on a preparative scale and stopped at 20% conversion (Table 2, entry 5) for the recovery (2.64 g/L) of an interesting pure tetrasaccharide (2, Figure 2) isolated from the reaction mixture by Biogel P-2 purification. The compound showed a peak in the MS spectrum at m/z 569 [M + Na]^+^ corresponding to a tetrasaccharide made of pentose sugars. ^1^H and ^13^C NMR signals are reported in Table 3. NMR signals of anomeric protons were found in the range 4.35-5.32 ppm and those of anomeric carbon atoms in the range 92.6-108.5 ppm. HMBC spectrum showed long-range correlations between ^13^C anomeric signal of α-L-Ara*f* and signal of H3 proton of central xylose and the related proton anomeric signal of α-L-Ara*f* and C3 of central xylose (see Table 3 for values). Suitable resolution of spectra for signal assignment of both compounds in Figure 2 was achieved only in dilute conditions (3–4 mg/ml); more concentrated samples furnished unresolved spectra and time-dependent signal shifts. Despite the structural reporter group concept for these structures is widely known (Vliegenthart et al. [1992]), structures in Figure 2 were established by analysis of ^1^H-^13^C NMR correlations on dilute samples.

Amount of xylose was formed also in the case of *G. thermantarcticus* (Table 2, entries 7–8) in accord to the loading of the enzyme. Using high amount of substrate per mg of crude biocatalyst (Table 2, entry 8) interesting oligomers accumulated (from xylobiose to other high molecular oligosaccharides, Figure 3).

Transglycosylation reactions were performed using *A. donax* RK72h biomass as donor and different biocatalysts (entries 9–12, Table 1). At the end of each reaction, total chromophoric xylosylated fraction was obtained by reverse phase column and analyzed by TLC with authentic standards (Tramice et al. [2009]), and MS spectroscopy. Mono- (m/z 351 [M + Na]^+^) and di-xylosylated (m/z 483 [M + Na]^+^) 9-fluorene methanol were identified in the reaction using *T. maritima* xylanase. The same reaction was performed using *T. neapolitana, T. thermostercoris* and *G. thermantarcticus,* crude homogenates. In the latter case mass peak at m/z 615 [M + Na]^+^ due to trixylosylated 9-fluorene methanol was also observed. Although not optimized for the yield, these reactions for the formation of the xylosylated products starting with *Arundo* biomass are of interest for enlarging the portfolio of pentose-specific conversion/utilization technologies (Dumon et al. [2012]).

### Biological activity

Samples of raw materials, extracts of rhizome and purified compounds from hydrolysis reactions were assayed for their antimicrobial, cytotoxic and antioxidant activities. Antimicrobial activity was evaluated on three bacterial strains, *E. coli* (DSM 498), *B. subtilis* subsp. *spizizenii* (DSM 347), and *M. luteus* (DSM 348). All samples did not show any significant antimicrobial activity. Brine shrimp assay was utilized as indicative of cytotoxicity (Meyer et al. [1982]). The brine shrimp is a marine crustacean used at stage of larvae (48 h after hatching). LD_50_ is the minimum lethal dose for which we observe a 50% mortality of larvae. Results obtained by using this test showed that all samples were not toxic on brine shrimp larvae; in fact after 24 h of exposure to samples at concentration of 100, 10 and 1 μg/ml, all larvae were live and motile.

Antioxidant activities of the raw material and of the extract of rhizome RK72h compared to that observed using fucoidan were good both in DMPD and DPPH assays. Inhibition of DMPD radical cation (43 and 35%) and DPPH radical (68 and 45%) were observed for raw material and extract of rhizome RK72h, respectively at the maximum tested amount (4 mg). Dose dependent activity was observed at lower concentrations (2 to 0.1 mg). Unfortunately purified compounds (Figure 2) did not exhibit significant antioxidant activity.

## Discussion

In this article the optimization of hemicellulosic substrate preparation from rhizome of *Arundo donax* has been reported. Hemicellulosic fraction represented ca. 20% of the dried biomass and occurred in a partially de-branched form after alkaline extraction, as indicated by Ara/Xyl ratio (Verbruggen et al. [1995]) and ^13^C NMR spectroscopy.

The production of two different interesting oligosaccharides was possible with both commercial (xylobiose, pentasaccharide 1) and thermophilic microorganisms (tetrasaccharide 2) from this hemicellulose fraction. ^1^H-^13^C NMR correlations of products were studied for precise structural assignment. Significant amounts of low molecular mass compounds, xylobiose and trace amounts of xylose were liberated from birch wood xylan after 24 h (Lin et al. [1999]) using *Thermomyces lanuginosus* enzyme; however cellulase activity could contaminate this endo-β-(1 → 4)-xylanase preparation (Subramaniyan and Prema [2002]) explaining the results obtained. Xylanase from *Thermotoga maritima* is an extremely thermostable enzyme which hydrolyzed xylo-oligosaccharides and xylans to yield predominantly xylobiose as end product (Jiang et al. [2004]). Xylobiose and small xylooligomers as main products in high amount from *Arundo* biomass confirmed this activity. The absence of glucose can be explained by better purity of enzymatic preparation (xylanase) with respect to *T. lanuginosus* reaction. As far as *T. neapolitana* CFE-TN and *T. thermostercoris* CFE-TT were concerned, presence of xylanases, xylosidases, glucuronidases and arabinofuranosidases were confirmed in the crude extracts by the action on suitable chromophoric and polysaccharidic substrates (data not shown). The results on *Arundo* indicated also that action of β-glucosidases is dependent on enzymatic load in the reaction mixture, and this phenomenon could be analyzed with respect to the known interrelation of cellulases and xylanases in the bioconversion of lignocellulosic material (Hu et al. [2011]). All these data are of great interest in the view of research for biocatalyst suitable for complete saccharification of the cellulose and hemicellulose components in raw material to fermentable sugars.

The production of the transxylosylated products from *Arundo* was also possible with thermophilic enzymes and this is of further interest for enlarging pentose-specific conversion/utilization technologies.

In conclusion rhizomes represent a useful raw material for the production of valuable industrial products increasing the economic and energetic efficiency of *A. donax* cultivation. Antioxidant activities of the raw material and hemicellulose fraction from rhizomes compared to that observed using fucoidan are good both in DMPD and DPPH assays.

## Competing interests

The authors declared that they have no competing interests.

## Authors’ contributions

LL conceived part of the study and VC carried out microbiological studies, ATra. and EP carried out the preparative enzymatic reactions, chromatographic purification and structural study of xylooligosaccharides, IF carried out the analytical enzymatic reactions, enzymatic assay and participated in the design of the study, GA and PdD carried out optimization of hemicellulose extraction from rhizome, MF is the responsible of the entire project and with MM and AI they were involved in the rhizome production and collection, GT carried out activity assay, BN and AP conceived part of the study and participated in the coordination, A. Tri. conceived part of the study, participated in the structural study of xylooligosaccharides and drafted the manuscript. All authors read and approved the final manuscript.
